# TaMoNbTiZr Multielement Alloy for Medical Instruments

**DOI:** 10.3390/ma18081876

**Published:** 2025-04-20

**Authors:** Ileana Mariana Mates, Victor Geanta, Doina Manu, Hajnal Kelemen, Adrian Emanuel Onici, Julia Claudia Mirza-Rosca, Ionelia Voiculescu

**Affiliations:** 1 “Dr. Carol Davila” Clinical Hospital of Nephrology, Carol Davila University of Medicine and Pharmacy, 010731 Bucharest, Romania; ileana.mates@yahoo.ro; 2 Material Science and Engineering Faculty, National University of Science and Technology Politehnica Bucharest, 060042 Bucharest, Romania; victorgeanta@yahoo.com (V.G.); onici.adrian@yahoo.ro (A.E.O.); 3 Centre for Advanced Medical and Pharmaceutical Research, University of Medicine, Pharmacy, Science and Technology “George Emil Palade”, 540142 Targu Mures, Romania; doina.manu@umfst.ro; 4 Department of Pharmaceutical Chemistry, Faculty of Pharmacy, University of Medicine, Pharmacy, Science and Technology “George Emil Palade”, 540142 Targu Mures, Romania; kelemen_h@yahoo.com; 5 Mechanical Engineering Department, University of Las Palmas de Gran Canaria, 35001 Las Palmas de Gran Canaria, Spain; julia.mirza@ulpgc.es; 6 Materials Engineering and Welding Department, Materials Science and Engineering Faculty, Transilvania University of Brasov, 500036 Brasov, Romania; 7 Industrial Engineering and Robotics Faculty, National University of Science and Technology Politehnica Bucharest, 060042 Bucharest, Romania

**Keywords:** biomaterial, heat treatments, microstructure, microhardness, biocompatibility

## Abstract

In this paper, a new TaMoNbTiZr multielement alloy has been designed, using chemical elements that exhibit extremely low bio-toxicity for the human body. The alloy was obtained by melting in vacuum arc remelting (VAR) equipment MRF ABJ 900 from high-purity chemical elements (99.5%) as mini-ingots having about 40 g weight each. The biocompatible alloys underwent changes in hardness after performing the annealing at 900 °C for 2 h, followed by cooling in water. The new alloy had an average hardness in the cast state of 545 HV0.5, and after heat treatment, it hardened to a value of 984 HV0.5, over 40% higher than that in the casting state, which ensures a longer working period. To use them as materials for medical instruments, their biocompatibility was highlighted through specific laboratory tests. For this, mesenchymal stem cells isolated from bone tissue and a human fibroblast cell line were cultured in vitro on the TaMoNbTiZr alloy’s surface. The biocompatibility of the alloy with the biological environment was evaluated by analyzing cell viability, adhesion, and proliferation, and in parallel, the cytolysis effects manifested by the increase in lactate dehydrogenase activity in the culture media were analyzed.

## 1. Introduction

The multielement alloys designed for medical applications must have good mechanical strength and low elasticity modulus, absence of contamination effects, lack of adverse reactions developed by the tissue with which they come into contact, ease of processing and shaping, and, finally, high biocompatibility [1,2]. The multielement alloys possess superior properties to classical metals, especially mechanical (high strength and hardness, good wear [3,4,5]) and corrosion resistance in various environments [6,7,8,9].

Recently, the refractory multielement alloys having equimolar chemical composition have attracted the interest of researchers due to their potential use for biomedical applications [9,10,11,12,13]. These alloys are also used in the medical field for the manufacture of orthopedic implants (hip, knee, and shoulder prostheses), tending to replace titanium alloys, Co-Cr alloys, or stainless steel. New opportunities for different compositions and microstructures are offered in this area, especially for concentrated complex alloys (CCAs) [14,15,16]. After the intense development of refractory alloys consisting of a single BCC (body centered cube), like the W-Nb-Mo-Ta and W-Nb-Mo-Ta-V alloy systems, the new multi-element alloys (MEA) have become interesting as biomedical materials. The main multi-elements were designed using transition metals from alloying systems such as Nb-Mo-Ta-W, V-Nb-Mo-Ta-W, Ta-Nb-Hf-Zr-Ti, Hf-Nb-Ti-Mo-Ta-Ti-Zr, and equi-atomic alloys like Hf-Mo-Nb-Ta-Ti-Zr. The tensile strength of theses alloys is very high (900–1600 MPa) and is accompanied by considerable compressive strength (more than 2000 MPa) [17,18].

From a biocompatibility perspective, most of these elements are considered biocompatible, except for vanadium. It was found that adding W to NbMoTa alloys results in better ductility and thermal stability characteristics. For example, the hardness of alloys such as NbMoTaW and VNbMoTaW was 4.46 and 5.25 GPa, respectively, and the yield strengths at 1873 K were 405 and 477 MPa [18]. Todai et al. consider that the TiNbTaZrMo alloy contains phases that are non-toxic and do not cause allergies [19]. Wang et al. [20] stated that, for medical applications, among common metallic biomaterials, the Ti-rich alloy system Ti-Nb-Ta-Zr, having a BCC structure, can be superior to other Ti-based metallic biomaterials.

The combination of Ti, Nb, Ta, and Zr is favorable for the formation of a single solid solution phase in these alloys if the appropriate chemical composition is obtained [21,22,23,24]. The highest corrosion resistance after hardening was recorded by alloys with concentrations of 20, 28, and 34 at.% Mo. Alloys with a concentration between 20 and 28 at.% Mo are susceptible to inter-crystalline corrosion (ICC) after annealing at 600–800 °C because they precipitate carbides (Mo_2_C and M_12_C) on the grain boundaries, which depletes the boundary zones of molybdenum. Increasing the concentration to 34 at.% Mo leads to precipitation of the γ phase throughout the microstructure, leading to increasing hardness [23,24,25]. Increasing the Zr concentration in (MoNbTaTi)_100−x_Zr_x_ alloys can intensify segregation phenomena on the interdendritic areas of this element, leading to unbalanced solidification and the formation of BCC2 phases rich in Zr and Ti [26].

Heat treatments applied to the high-entropy alloys can result in either hardening effects or an increase in plasticity and toughness, depending on the heating temperature, the holding time, and the cooling mode. Thus, it was found that homogenization annealing combined with rapid cooling obtain high values of mechanical properties [27,28]. Through appropriate sequences of heat treatments (annealing and quenching in water, followed by aging), stabilization of the β phase in HfMo_x_NbTaTiZr alloys can be achieved for concentrations of this element above 10 wt.% Mo and high cooling rates [29]. Annealing heat treatments can reduce or eliminate the segregation effects of the chemical elements, which occur during the casting operation in the case of high-entropy alloys [28]. This results in microstructures closer to equilibrium, either by dissolving metastable phases or by nucleating equilibrium phases, which were prevented from forming during rapid cooling [29,30]. An increase in the annealing temperature above 1150 °C in molybdenum alloys is accompanied by a uniform reduction in yield strength, a characteristic that was observed at the annealing temperature corresponding to the primary recrystallization range. The dendritic morphology specific to cast alloys is not affected if the holding temperature value does not exceed 1040 °C [31,32]. On the contrary, when the temperature exceeds 1200 °C, phases rich in certain chemical elements appear. In the case of HfMoTaTiZr and HfMoNbTaTiZr alloys, a fracture strain of 12% has been obtained successfully at room temperature [33,34].

Some alloys, like FeMoTaTiZr, WNbMoTa, and WNbMoTaV, were tested for various uses in the medical field (surgery or medical instruments) to improve the physical–mechanical characteristics and the biocompatibility [14,19,35]. To assess biocompatibility, standardized tests must be performed, such as electrochemical characterization in a simulated biological environment, cell viability assessment, cytotoxicity assessment, sensitization, irritation or subcutaneous reaction test, acute and subacute systemic toxicity, chronic and sub chronic systemic toxicity, genotoxicity, and implantation [36,37,38,39]. In vitro studies commonly use bone marrow-derived mesenchymal stem cells (BDMSC) to analyze the biocompatibility of certain replacement biomaterials used in implantology [40,41].

Osteomorphogenesis is dependent on the cell’s attachment, adhesion, and spreading on a biomaterial substrate, which must be non-toxic and favorable for cell viability and functioning [42,43]. A commonly used method for determining cytotoxicity is based on measuring the activity of cytoplasmic enzymes released by damaged cells. Lactate dehydrogenase (LDH) is a stable cytoplasmic enzyme found in all eukaryotic cells. LDH is rapidly released into the culture medium when the plasma membrane of cultured cells is damaged in apoptosis, necrosis, and other forms of cell damage. The increase in LDH activity in the cell supernatant is proportional with the number of damaged cells [44,45].

In this paper, a new TaMoNbTiZr multielement alloy was obtained in vacuum arc remelting equipment, and the microstructure was characterized by SEM microscopy and XRD analysis. The hardness of the alloy increased substantially (with over 40%) by annealing at 900 °C, followed by water quenching. The high hardness of the alloy in the hardened state, double that of martensitic stainless steels, gives the sharp edges more wear resistance and longer maintenance of cutting ability. The evaluation of cell viability and proliferation provided by bone surgery is also presented, namely the evaluation of direct cell adhesion and degree of survival on the surface of the TaMoNbTiZr alloy. Mesenchymal stem cells showed good viability and proliferated to a confluence of 80–90% during the 10-day analysis, both in the case of metal-free cultures and in those containing samples of the analyzed alloy.

## 2. Materials and Methods

The TaMoNbTiZr multielement alloy was obtained in MRF ABJ 900 Vacuum Arc Remelting (Allenstown, NH, USA) equipment from the ERAMET Laboratory of Politehnica University of Bucharest. The alloy’s recipe was designed based on the chemical elements’ effects and the previous results of the team [12,38]. The raw materials were high-purity chemical elements (purity higher than 99.3%) that were first cleaned and weighed before introduction into the RAV installation. For obtaining the right proportion of chemical elements required by the high-entropy alloy definition, the raw materials were dozed for the achievement of the recipe ratios.

The melting was performed under an argon atmosphere, with the electric arc formed between the tungsten electrode and the raw materials placed on the cells in the water-cooled copper plate of the installation. To ensure a low oxygen level in the melting chamber, argon flushing and successive vacuuming were performed using the preliminary vacuum pumps, and then, the diffusion pumps were connected to reach the required vacuum level of 5 × 10^−3^ mbar. Subsequently, the working chamber was flooded with a high-purity argon flow (Argon 5.3) under a pressure of 2.5 bar for 20 min to generate the protective atmosphere during melting and to ensure the stability of the electric arc burning.

**Figure 1:**
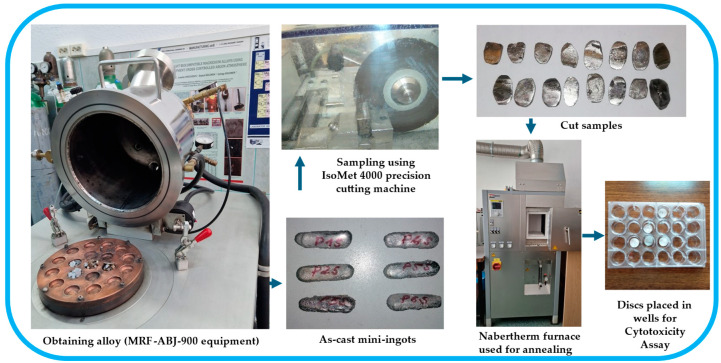
Flowchart of the activities performed to obtain and test the TaMoNbTiZr alloy.

Based on the atomic mass values of the constituent chemical elements (M_Ta_ = 181 g/mol; M_Mo_ = 96 g/mol; M_Nb_ = 93 g/mol; M_Ti_ = 48 g/mol; M_Zr_ = 91 g/mol), the total molar mass of the ingot was 509 g/mol. Thus, for a ingot of 40 g, the quantities of materials used were as follows (g): Ta = 14. 22; Mo = 7.54; Nb = 7.31; Ti = 3.77; Zr = 7.15. The resulting percentage composition was the following (wt.%): Ta = 38.95; Mo = 17.32; Nb = 13.07; Ti = 13.21; Zr = 17.45. To obtain good homogeneity of the alloy, successive melts were performed, 8–10 times on each side of the mini-ingots. The mini-ingots were then cut and machined to obtain 1 mm thick discs using the precision cutting machine IsoMet 4000 (Buehler, Leinfelden-Echterdingen, Germany). Then, some of the discs were annealed at 900 °C for 2 h in an argon atmosphere in a Nabertherm furnace (Lilienthal, Germany) and then cooled in water. The flowchart of the technological research activity is shown in Figure 1.

The samples taken from the ingots were prepared by the metallographic procedure according to [46] (grinding with abrasive papers with progressive grain size from 400 to 1200, polishing with alpha alumina powders in suspension with grain size of 0.1 microns). The microstructure characterization of the new alloy was performed using a field emission scanning electron microscope FESEM-FIB (Auriga—Carl Zeiss SMT, Oberkochen, Germany) equipped with a Gemini column for the electron beam. For the compositional analysis, the SESI secondary electron detector of the Everhart Thornley type with Faraday cup in the sample chamber for SEM imaging was used, as the X-Max detector produced by Oxford Instruments ltd. with an SDD type active element for energy dispersive spectral analysis for elemental mapping and surface composition. X-ray diffraction analysis was performed using a D8 Discover diffractometer (Bruker-Berlin, Germany), configured on the primary optics with a Cu primary radiation tube (λ = 1.540598 Å), a Göebel mirror, and on the secondary optics with a 1D LynxEye detector. The diffractograms were recorded with an angular increment of 0.040 at a scanning speed of 1 s/step. Qualitative analysis was performed using the ICDD Release 2014 database.

The microhardness measurement was performed both on as-cast and heat-treated alloys according to [47] using a Shimadzu HMV 2T apparatus (Shimadzu, Kyoto, Japan) by setting a force value of 4.903 N and an indentation time of 10 s.

In this study, mesenchymal stem cells isolated from bone tissue (HBMSC) and a human fibroblast cell line (NHF) kindly provided by the Angiogenesis and Vascular Biology group at Manchester Metropolitan University were cultured in vitro in the presence of TaMoNbTiZr alloy discs to evaluate biocompatibility.

The bone tissue used for isolation was removed from the hip joint, from the open end of the femoral head, before the insertion of the femoral prosthesis during the total arthroplasty operation at patients with osteoarthritis. The patients signed the informed consent, according to the agreement of the Medical Ethics Committee of the Targu Mures (Romania) County Emergency Hospital, no. 19263/31.07.2017. The bone tissue fragments were transferred to Dulbecco’s modified minimal essential medium (DMEM, Sigma cat. No. D6046, St. Louis, MO, USA) supplemented to a final concentration of 1% with penicillin/streptomycin (Sigma cat. no. P4333) and 10% fetal bovine serum (FBS) (Sigma F7524) and centrifuged at 4000 rpm for 10 min to dislodge the stem cells. Bone fragments were cultured as explants (E1) in a 75 cm^2^ flask (Eppendorf cat. no. 0030711122, Hamburg, Germany) at 37 °C in a humidified atmosphere with 5% CO_2_. The explant cultures were left in the incubator for 7 days, after which, the medium was replaced. Stem cells reached confluence after 3–4 weeks, and for the biocompatibility experiments, we used HBMSC in passage 2 (P2) [48].

After surface preparation (polishing with 600-grit abrasive paper, washed with alcohol, dried with hot air), the alloy samples were placed in 24-well plates (Eppendorf, cat. no. 0030722116) after UV sterilization. A total of 3 × 10^4^ NHF and 3 × 10^4^ HBMSC were seeded on the surface of each disc. After a 30 min period for initial cell adhesion, 1 mL of supplemented Dulbecco’s modified minimal essential medium (DMEM) was added. Once a certain seeding number of each cell type was established, the protocol was kept unchanged throughout the experiments.

Cells in direct contact with the alloy samples were analyzed with a Leica DMi8 inverted microscope (Leica Microsystems CMS GmbH, Mannheim, Germany) in fluorescence using dedicated filters, Fluorescein isothiocyanate and Rhodamine fluorescence cubes, after incubation with the Live/Dead Cell Double Staining Kit (Sigma cat. no. D4511-1KT-F) containing fluorochromes Propidium Iodide (PI) and Calcein AM. The excitation source of the fluorochromes (Calcein AM and PI) was a Leica EL6000 lamp, and image acquisitions were performed with a Leica DFC 450C digital camera. Next, the phase contrast image acquired on the same microscopic field of view (FOV) allowed for easy visualization of the alloy sample in the well.

Cell viability and proliferation and direct cell adhesion on the alloy surface were evaluated for 5 days and 10 days. Cytolysis was evaluated by measuring the lactate dehydrogenase (LDH) activity in the culture media. Biological media (cleared by centrifugation before testing) were analyzed in duplicate for LDH using the Cobas Integra 400 Plus biochemical analyzer (Roche Diagnostics, Block Scientific Inc., Sawgrass Drive, Bellport, NY, USA) [49].

## 3. Results

### 3.1. Microstructure

**Figure 2:**
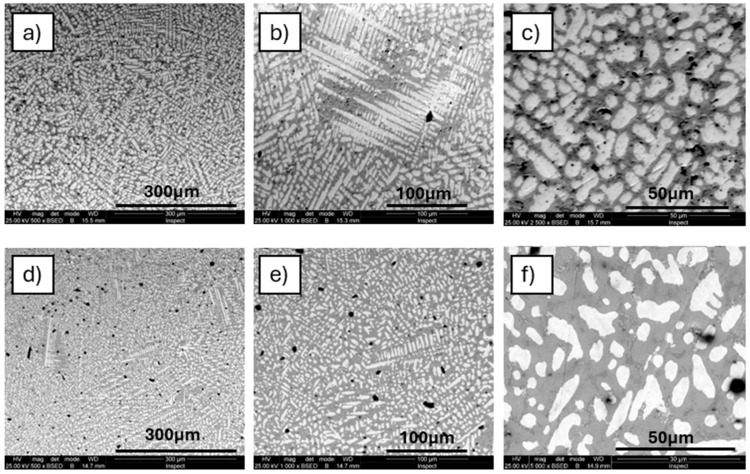
SEM microstructure of TaMoNbTiZr alloy: (**a**–**c**) as-cast; (**d**–**f**) annealed at 900 °C for 2 h, followed by quenching in water.

The microstructure analysis of the new multielement TaMoNbTiZr alloy was performed before and after heat treatment to evaluate the changes induced by it. The purpose of the heat treatment was to reduce internal stress, to obtain better homogenization of the chemical composition, and to increase the hardness, desired for the manufacture of cutting blades of surgical knives. The changes in the size and shape of the dendrites in the alloy microstructure after heat treatment can be observed in Figure 2.

**Figure 3:**
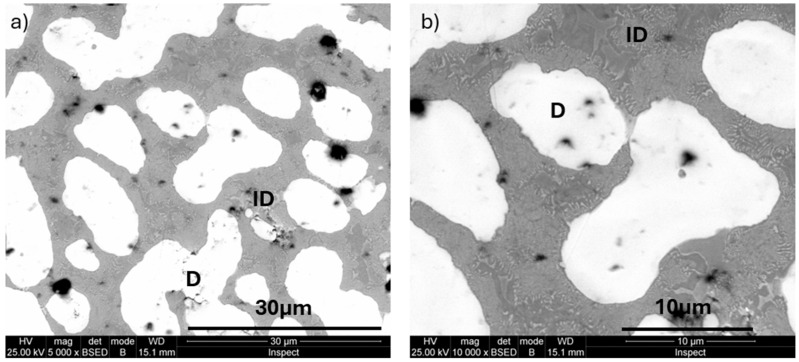
Detail on annealing samples at different magnifications: (**a**) ×5000; (**b**) ×10,000 (D—dendrite; ID—interdendritic zone).

The images of the microstructure observed at progressive magnifications (Figure 2a–c, or the as-cast state and Figure 2d–f for the annealed state) highlight the thinning and rounding of the dendritic branches and a general grain refinement effect after heat treatment. At high magnification (×5000 and ×10,000), in the interdendritic zone (Figure 3), the effect of dendrite rounding (D) and the separation of secondary phases in the interdendritic zone (ID) can be observed more clearly.

**Figure 4:**
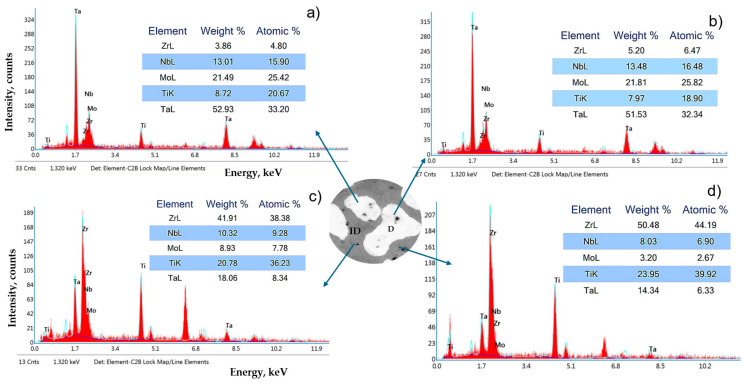
Semi-quantitative composition spectra of the integrated areas in Figure 3b. (**a**,**b**) composition for dendritic zone; (**c**,**d**) composition for inter-dendritic zone.

The new secondary phases that appear in the interdendritic zone after annealing were highlighted by XRD analyses. To assess the distribution of elements within the metallic matrix, local chemical micro-composition analyses were performed on dendrites and interdendritic areas using the EDAX method (Figure 4).

**Figure 5:**
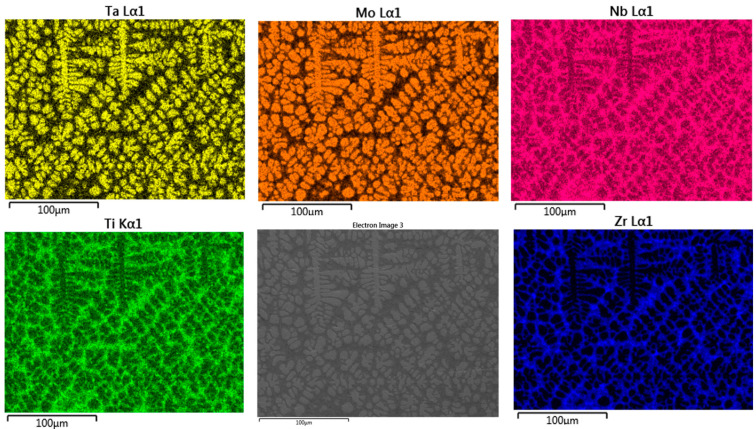
Element distribution map in the microstructure of the as-cast TaMoNbTiZr alloy.

It was observed that elements such as Mo and Ta are preferentially distributed in dendrites, while Ti and Zr have the highest concentrations in the interdendritic areas. Only Nb has a quasi-equal distribution in both regions, with slightly higher concentrations into dendrites. The element distribution map in the microstructure of the multicomponent MoNbTaTiZr alloy after casting highlights a homogeneous dendritic structure, with the alloy constituents being clearly differentiated (Figure 5).

**Figure 6:**
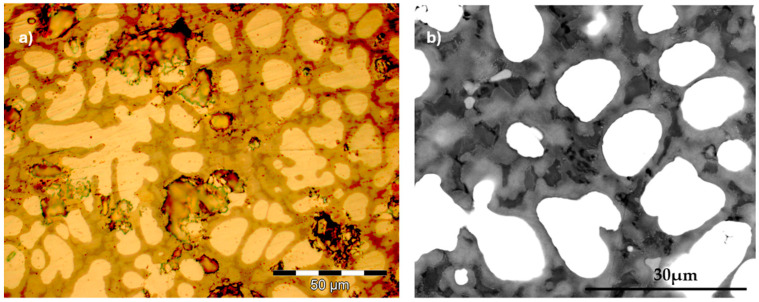
Oxidation of interdendritic areas during heat treatment of TaMoNbTiZr alloy: (**a**) optical image; (**b**) SEM Image.

Annealing at 900 °C for 2 h, followed by water quenching, contributed to the homogenization of the alloy and the dissolution of unstable constituents. At the same time, two new secondary phases appeared, which contributed to the reinforcement of the metal matrix. Since Ti and Zr in the alloy recipe are very reactive to oxygen, even though a protective atmosphere was used in the heat treatment furnace, oxidation effects (brown and black area) appeared, especially in the interdendritic areas where the proportion of these elements is higher (Figure 6).

**Figure 7:**
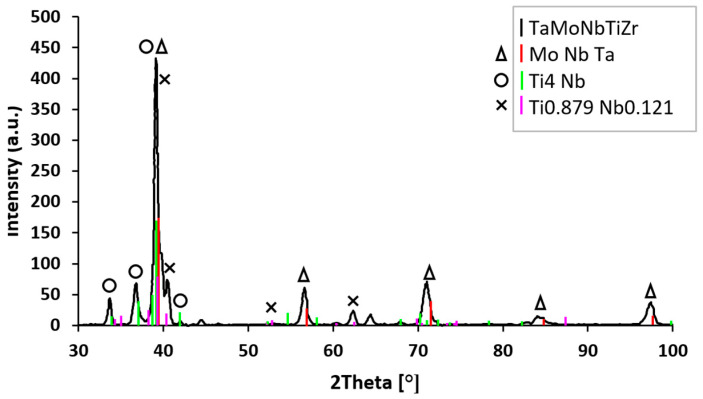
X-ray diffractogram recorded for as-cast TaMoNbTiZr multicomponent alloy.

To identify the compounds’ lattice parameters in the alloy, the initial structural characterization performed by X-ray diffraction was refined using the Rietveld technique and TOPAS program. The X-ray diffractogram of the TaMoNbTiZr multicomponent alloy is presented in Figure 7. Along with the TaMoN TiZr solid solution, three more phases appear, namely MoNbTa, Ti_4_Nb, and Ti_0.879_Nb_0.121._

**Figure 8:**
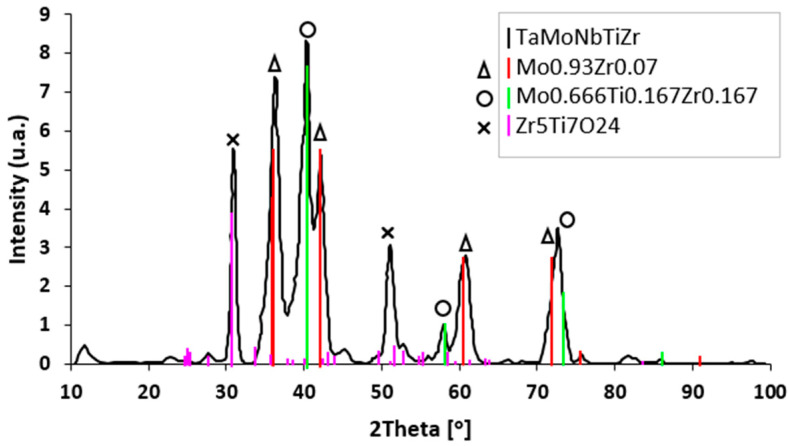
X-ray diffractogram recorded after heat treatment of TaMoNbTiZr alloy.

Diffraction analyses performed after performing the heat treatment (Figure 8) revealed the formation of a complex oxide type (Zr_5_Ti_7_O_24_), which was due to the heating atmosphere (air) and cooling medium (water). At the same time, new phases (Mo_0.93_Zr_0.07_, Mo_0.666_Ti_0.167_Zr_0.167_) were identified. The dissolution of existing unstable phases in the cast alloy (Ti_4_Nb, Ti_0.879_Nb_0.121_) occurred because of the diffusion of chemical elements during heat treatment. These elements migrate by diffusion to form more thermodynamically stable compounds. The formation of complex oxides with a highly ordered structure and the associated work hardening caused a change in the plastic deformation mode of the metallic matrix, from planar sliding (typical of classical alloys) to wavy sliding [50,51].

W. Lai et al. [52], who studied three alloys from the Ti-Zr-Nb-Ta-Mo system, found that, during the solidification of the alloys, secondary phase separation (even at the atomic scale) occurred due to miscibility gaps. By changing the concentrations of Ta and Nb, they found that the phase separation effect was influenced by both alloying elements, but the most pronounced effect was with increasing the concentration of Ta. Also, Luo X. et al. [53] observed that a redistribution of chemical elements occurred after solid solution treatment compared to the as-cast state. The cumulative effects of secondary phase separation, the formation of complex oxides, the cocktail effect generated by the association of chemical elements with atomic diameters that present notable differences, the multiplication of dislocations, and the blocking of their movement by grain boundaries can be causes that generate the significant increase in hardness after annealing treatment.

### 3.2. Microhardness

The microhardness values measured on the experimental samples of the TaMoNbTiZr alloy in the as-cast state and after performing heat treatment are presented in Table 1. The samples for hardness measurement were extracted from both the as-cast and heat-treated samples. Their cutting was performed under cooling liquid using a precision cutting machine. After cutting, the surfaces were ground with abrasive papers and then polished with a suspension of alumina powders following the same procedure as for the metallographic study. For each sample, sets of five measurements were performed in directions perpendicular to the axis of the ingot, maintaining distances between indentations of at least 500 microns.

### 3.3. Biological Tests

**Figure 9:**
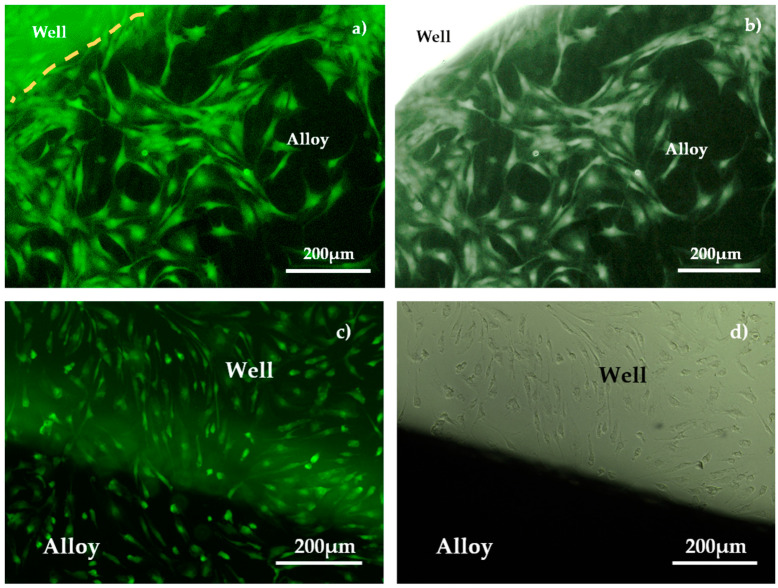
Viability, adhesion, and proliferation of HBMSC on TaMoNbTiZr alloy: (**a**) fluorescence and (**b**) phase contrast for same field of view after 5 days; (**c**) fluorescence and (**d**) phase contrast after 10 days for same field of view.

**Figure 10:**
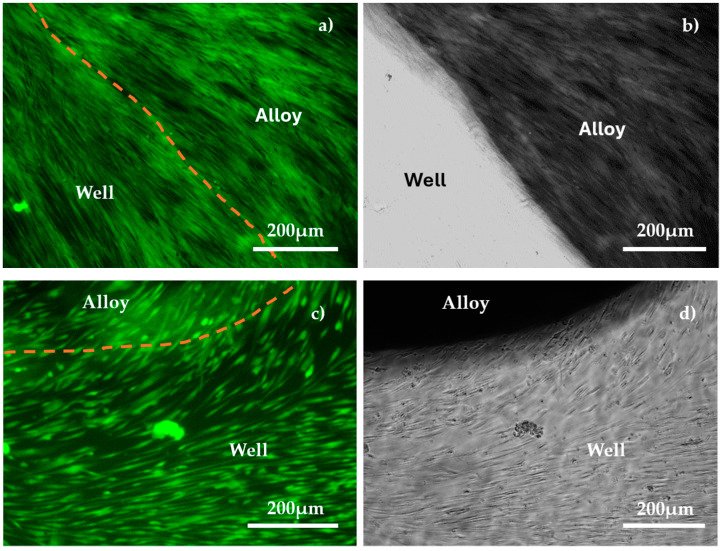
Viability, adhesion, and proliferation of human fibroblast cell line upon contact with alloy: (**a**) fluorescence and (**b**) phase contrast after 5 days; (**c**) fluorescence and (**d**) phase contrast after 10 days for same field of view.

Cell viability, proliferation, and direct cell adhesion on the alloy surface were tested twice during the experimental procedure, after 5 days and 10 days, respectively. The analysis of the alloy’s biocompatibility was performed by qualitatively evaluating cell viability and proliferation using optical microscopy (Figure 9) as well as by assessing cytotoxicity effects, determining lactate dehydrogenase activity levels in the culture media (Figure 10).

#### 3.3.1. Biocompatibility Test

Calcein AM is a cell-permeable dye used for live-cell labeling. Once inside the cell, intracellular esterases from living cells remove the acetoxymethyl (AM) ester group, converting it into the fluorescent Calcein dye, which cannot cross the cell membrane. In contrast, apoptotic and dead cells with damaged membranes are unable to retain Calcein. Fluorescent Calcein emits in the green fluorescence channel, making the HBMSC visible in indirect contact with the alloy and also those proliferating in the well (Figure 9). A similar evolution can be observed when using NHF (Figure 10).

From Figure 9 and Figure 10, both HBMSC and NHF are uniformly distributed over the entire surface of the samples, with no differences identified between the metal alloy or well areas for samples that were immersed for 5 days in biological solution. After 10 days of immersion, biological cell overpopulation of the metal sample surfaces appears, and the level of LDH increases. Because of this, the images in fluorescence and in phase contrast are unclear on the metal surface (they appear as dark or bright areas).

#### 3.3.2. Cytotoxicity Evaluation

**Figure 11:**
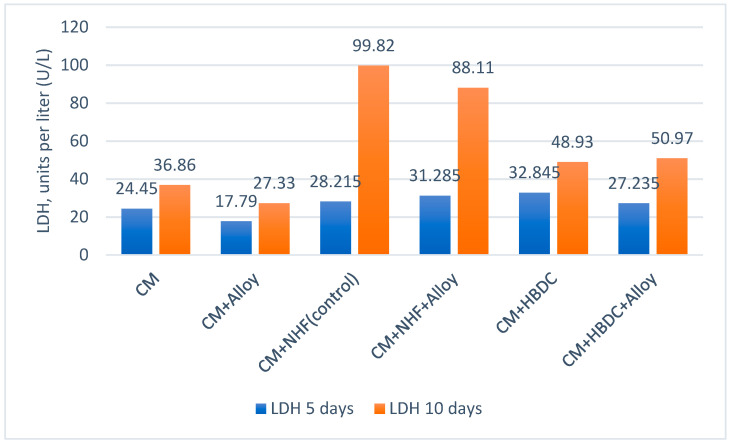
Comparative evaluation of LDH activity from culture media and MoNbTaTiZr alloy: CM—Culture media; NHF—Normal human fibroblast; HBDC—Bone mesenchymal stem cells; LDH—Lactate dehydrogenase.

Cytotoxicity tests aim to quantify LDH activity in the culture medium. LDH activity was determined by the UV enzymatic method. To assess the degree of cellular cytolysis, the levels of LDH were analyzed comparatively from cell culture media in the presence or in the absence of metallic material for both HBMSC and NHC. The results are presented in Figure 11.

Pronounced increases in LDH were recorded only in the NHF cultures, both on the surfaces of the experimental alloys and without the presence of the alloy. This trend indicates that the cell density in the analyzed solution was high enough to induce cytolysis to the wells without the alloy (88.11 (U/L). The presence of the metal further increased the LDH levels to 99.82 (U/L), which means that, after 10 days of immersion, more pronounced cellular cytolysis effects appear in the presence of the metal alloy.

## 4. Discussion

### 4.1. Microstructure Analysis

Both in the initial as-cast state (Figure 2a–c) and after heat treatment (Figure 2d–f), the TaMoNbTiZr alloy has a dendritic microstructure, but important differences can be observed in the size and shape of the dendrites. An important effect after annealing treatment is the finishing, thinning, and rounding of the dendritic branches. The material thus became more homogeneous and compact, and the formation of secondary phases generated a localized internal stress, which determined the consolidation of the metallic matrix.

The decomposition effects of secondary phases can occur at both the micro and atomic scales and have a significant impact on the mechanical properties of alloys in the Ti-Zr-Nb-Ta-Mo system. Since the secondary phases are randomly distributed in the metal matrix, heterogeneous deformation of the matrix occurs, which causes stress concentration at the grain boundaries [54].

EDS microchemical composition analyses performed on the heat-treated alloy show, in dendrites (D, Figure 3), high concentration values for Ta (51.53–52.93 wt.%) and Mo (21.49–21.81 wt.%), which means that they primarily participate in the formation of dendrites (Figure 4). The other elements in the D-zone have lower concentrations than prescribed for this alloy, respectively: Ti (7.97–8.72 wt.%) and Zr (3.86–5.20 wt.%). In this area, only Nb has a chemical concentration like the designed one, namely Nb (13.01–13.48 wt.%). The chemical composition of the interdendritic zones (ID) is very different from that of the dendrites. Here, the maximum concentration is reached by Zr (41.91–50.48 wt.%), followed by Ti (20.78–23.95 wt.%), Ta (14.34–18.06 wt.%), Nb (8.03–10.32 wt.%), and Mo (3.20–8.93 wt.%).

Regarding the type of phases that appear in TaMoNbTiZr system alloys, there is still no model that can encompass all the experimental results. Since it is a new alloy, research must continue to establish the best manufacturing recipes.

Several alloy variants are being studied, such as Ti_35_Zr_35_Nb_15_Ta_10_Mo_5_, Ti_35_Zr_35_Nb_10_Ta_15_Mo_5_, and Ti_32.5_Zr_32.5_Nb_15_Ta_15_Mo_5_, which show ultra-high tensile and yield strengths exceeding 1 GPa [52]. Although they contain the same chemical elements, there are large differences in the concentrations of the chemical elements, titanium being the element with the highest percentage. Therefore, it is not possible to predict the types of phases that may appear based on these alloys.

Zhou, Y. et al. [55] studied the TaMoNbZrTi alloy obtained by multi-wire arc additive manufacturing. The results of EDS chemical composition analysis were as follows (at.%): in the dendrite region, Ta 23.39 ± 1.19, Mo 38.72 ± 1.28, Nb 11.27 ± 0.44, Zr 11.18 ± 0.44, and Ti 15.44; ± 0.44; in the interdendritic region, Ta 4.96 ± 0.41, Mo 16.90 ± 0.72, Nb 6.89 ± 0.36, Zr 52.55 ± 2.12, and Ti 18.70 ± 0.33. Like in our study, they found that Ta, Mo, and Nb first form the BCC_B2#1 phase, while Zr and Ti participate preferentially in the formation of the secondary BCC_B2#2 phase. For better comparison, in our study, the following concentrations were recorded (at.%): in the dendrite region, Ta 32.34 ± 1.2, Mo 25.62 ± 0.20, and Nb 16.29 ± 0.19; in the interdendritic region, Ta 7.33 ± 0.11, Mo 5.17 ± 2.5, Nb 8.05 ± 1.11, Zr 41.28 ± 2.9, and Ti 38.06 ±1.85. Considering the different conditions of obtaining alloys (alloy recipe, melting and cooling rate, applied heat treatments), differences in the concentration of chemical elements in dendritic or interdendritic areas are evident. However, similarly higher values of the concentrations for the elements Mo, Ta, and Nb are observed in dendrites and for Ti and Zr in interdendritic areas.

From Figure 5, it can be clearly observed that Mo and Ta are predominantly present in the dendrite constitution, a fact confirmed by the structural characterization of the studied samples, while Ti and Zr are predominantly present in the interdendritic zone. The presence of Nb in both zones of this alloy, with concentrations between 8 and 13 wt.%, led to the grain refinement and the reduction in interdendritic porosity, an effect also known from the specialized literature [39,51].

Although the effect of heat treatment was beneficial in terms of increasing hardness and homogenizing the chemical composition of the alloy, undesirable oxidation effects appeared on the interdendritic area (Figure 6). Therefore, for these alloys it is recommended to perform heat treatments in a controlled atmosphere, and if possible, cooling must be performed in an argon flow [12].

From the X-ray diffractogram of the multicomponent alloy TaMoNbTiZr presented in Figure 7, the following components are found: MoNbTa, Ti_4_Nb, and Ti_0.879_Nb_0.121_. The MoNbTa compound, identified at an angle 2θ = 39°, is the most prominent. Very close to it, also at an angle 2θ of approx. 39°, are the compounds Ti_4_Nb and Ti_0.879_Nb_0.121_. This composition is in good agreement with the designed recipe, with only minor alloy losses during processing. From the X-ray diffractogram, the main phase is Mo-Nb-Ta, with a crystal structure b.c.c. (body-centered cubic), that has the lattice parameter a = 3.24 (9) (Å) and crystallite size of 16.1 (nm). Another compound is Ti_4_Nb, with lattice parameters (Å) a = 3.19 (8), b = 4.87 (5), and c = 4.60 (7); crystallite size of 102 (nm); and an orthorhombic crystal structure. Also present is the phase Ti_0.879_Nb_0.121_ that has the lattice parameters (Å) a = 3.11 (9), b = 5.09 (7), and c = 4.62 (1); crystallite size of 81 (nm); and the same orthorhombic spatial group.

The homogenization of the solid solution was achieved by annealing and quenching in water, and the compounds that were separated from the Mo-Nb-Ta b.c.c. solid solution are Mo_0.93_Zr_0.07_, with lattice parameter of a = 4.31 (Å), crystallite size of 5.6 (nm), and f.c.c. (face-centered cubic) crystal structure; Ti_1.83_Zr_0.17_, with lattice parameters of a = 2.82 (Å) and c = 4.73 (Å), crystallite size of 2 (nm), and hexagonal crystal structure; Mo_0.666_Ti_0.167_Zr_0.167_, with lattice parameter of a = 3.19 (Å), crystallite size of 5.7 (nm), and b.c.c. crystal structure; and Zr_5_Ti_7_O_24_, with lattice parameters of a = 15.34 (Å) and c = 5.02 (Å), crystallite size of 12 (nm), and an orthorhombic crystal structure.

### 4.2. Microhardness Evolution

As the data obtained show, the biocompatible alloys underwent hardness changes following the annealing treatment at 900 °C for 2 h, followed by water quenching. In the as-cast state, the alloy had an average hardness of 545 HV0.5, and after the heat treatment, it hardened to a value of 984 HV0.5. The increase in the alloy hardness is over 40% compared to the value obtained in the as-cast state. Oxygen can be used as a dopant in certain multicomponent alloys such as TiZrHfNb [50,51]. The presence of oxygen in the metal matrix as compounds led to a yield strength of 1300 MPa and an elongation of 30% at room temperature. In our alloy, this effect was generated by the formation of new compounds with high hardness and thermal stability, such as Mo_0.666_Ti_0.167_Zr_0.167_. Also, the compound Zr_5_Ti_7_O_24_ appeared due to oxidation during heat treatment, being characterized by a crystal parameter of the atomic network three times larger than that of the metallic matrix (a = 15.34 (Å)). The formation of this compound determined the local distortion of the crystal network, which induced localized internal stresses and hardness increases.

It is known that, in solid solution alloys, lattice distortions are generated by mutual interactions between atoms with different radii or moduli, leading to the development of localized elastic stress fields. However, solid solution strengthening of multi-principal element alloys assumes a completely random elemental distribution, which does not agree with the real elemental distribution in the crystal structure of alloys such as Ti-Zr-Nb-Ta-Mo, characterized by dual-scale phase decomposition [52,53].

In the case of Ta-Mo-Nb-Ti-Zr alloys, it is not sufficient to apply the conventional model for evaluating the strengthening effect based on theoretical calculations of the strength of the dendritic (DR) arms, interdendritic (IDR) region. If the consolidation effect of the grain boundaries is not considered, mechanical strength values lower than those determined through practical experiments will be obtained. Atomic-scale phase separation in this alloy can promote more complex mechanisms at the level of the metal matrix, causing severe distortions of it, with the consequence of increasing the strength of the alloy by reducing the mobility of dislocations due to the blocking of their movement and blocking along the consolidated grain boundaries.

Q. Li et al. [54] observed that the decomposition effects of secondary phases occur at both the micrometric and atomic scales and have a significant impact on the mechanical properties of alloys in the Ti-Zr-Nb-Ta-Mo system. Since the secondary phases are randomly distributed in the metal matrix, heterogeneous deformation of the matrix occurs, which causes stress concentration at the grain boundaries.

Although it has been theoretically assumed that the presence of interstitial O atoms can cause charge transfer and lattice distortion, it is difficult to establish a precise model for estimating the influence of oxygen on the elastic properties of multi-principal element alloys. Recent studies have shown that, with the increase in the doping content of interstitial O and N atoms, more obvious lattice distortions appeared in the TiZrHfNb system alloys simultaneously with the increase in the lattice parameters and the decrease in their density [54].

### 4.3. Biocompatibility Analysis

#### 4.3.1. Biological Evaluation

The HBMSC were viable and maintained good adhesion to the substrate, both on the surface of the metal alloy and on the test well, after 5 or 10 days of immersion in the test solution (Figure 9c,d). Figure 10a,b, indicates that the NHF has good proliferation and adhesion tendency to both the TaMoNbTiZr alloy disk and the test well after 5 days of maintenance. By increasing the immersion period to 10 days (Figure 9c,d), an increase in cell dimension but a reduction in their number for the same observation area were observed. Higher values of LDH activity were found in the wells in which NHF was cultured, probably due to the more intense proliferation of this cell line. One of the causes of apoptosis and necrosis occurring in vitro cell cultures is the overcrowding of the culture surface with cells. Cell passages in culture should be performed in the logarithmic phase of cell growth, without allowing a confluence exceeding 80–90%. In the analyzed wells, NHF reached a confluence of 100%, with areas of overlapping (clumping) of cells in the well after 10 days of contact with the alloy.

#### 4.3.2. Cytotoxicity Evaluation

From the data presented in Figure 11, a slight increase in LDH activity was recorded after 10 days, both in the control wells in which the cells were cultured without alloy and in the wells in which the cells were cultured in the presence of alloy. The increase in LDH activity in both situations demonstrates that certain cytolysis phenomena occurred even in the absence of alloy. Higher values of LDH activity were observed in the wells cultured with NHF, probably due to the more intense proliferation of this cell line. A possible cause of the occurrence of apoptosis and necrosis in in vitro cell cultures can be determined by the overcrowding of the culture surface with cells at over 80–90%. In the analyzed wells, NHF reached 100% confluence, with areas of overlapping (overcrowding) of cells in the well after 10 days of contact with the alloy. Lower LDH increases were recorded in the presence of the metal alloy compared to the case when only NHF cells were tested in the same culture medium. When the alloy sample was introduced into the culture medium with HBMDC, the amount of LDH increased slightly after 10 days of maintenance from 48.93 to 50.97 units per liter (U/L).

## 5. Conclusions

In this study, research was conducted to obtain and test a new TaMoNbTiZr multielement alloy intended for the manufacture of medical instruments or sampling organic materials. Chemical elements with high biocompatibility, used globally for the manufacture of medical devices, were chosen for the recipe of the new alloy.

Microstructural analyses revealed the formation of a dendritic microstructure, with preferential localization of Ta (32.34 at.%) and Mo (25.62 at.%) in dendrites, while Ti (38.06 at.%) and Zr (41.28 at.%) were concentrated in interdendritic areas. Niobium had a quasi-uniform distribution tendency, with higher concentrations in dendritic areas (16.29 at.%) and medium concentration in interdendritic areas (8.05 at.%).

At the same time, the formation of a complex oxide of the reactive elements Zr and Ti, with a mass proportion of about 36%, is noted. The formation of this compound can be limited by performing the treatment in a furnace with a controlled argon atmosphere and cooling under argon flow.

The annealing heat treatment performed at 900 °C for 2 h, followed by quenching in water, determined the separation of new phases (Mo_0.93_Zr_0.07_, Mo_0.666_Ti_0.167_Zr_0.167_), different from those that appeared in the as-cast state (Ti_4_Nb, Ti_0.879_Nb_0.121_), which contributed to the consolidation of the metal matrix. After annealing, a significant increase in hardness was obtained from 545 HV0.5 (as-cast) to 984 HV0.5.

Biocompatibility and cytotoxicity tests showed that mesenchymal stem cells exhibited good adhesion to the metal surface, good viability, and proliferation up to a level of 80–90%. Adverse cytotoxicity effects were recorded only for maintenance periods of more than 10 days. Lactate dehydrogenase levels indicated normal apoptosis and necrosis effects in in vitro cell cultures, with and without the presence of the alloy.

Consequently, the alloy may be a good candidate for the manufacture of medical instruments.

## Figures and Tables

**Table 1:** Microhardness HV_0.5_ of TaMoNTiZr alloy.

Alloy	Individual Values					Average Value	Coefficient of Variation
As-cast state							
TaMoNbTiZr	515	535	565	564	546	545	3.85
Annealed and quenched							
TaMoNbTiZr	986	941	999	998	994	984	2.48

## Data Availability

The original contributions presented in the study are included in the article, further inquiries can be directed at the corresponding author.

## Abbreviations

The following abbreviations are used in this manuscript:

MEA	Multielement alloys
b.c.c.	Body-centered cubic
f.c.c.	Face-centered cubic
HBDC	Bone mesenchymal stem cells
FBS	Fetal bovine serum
LDH	Lactate dehydrogenase
NHF	Normal human fibroblast
DMEM	Dulbecco’s modified minimal essential medium
HBMSC	Human bone mesenchymal stem cells
CM	Culture media
FOV	Field of view
HV	Vickers hardness
